# Wide-genome selection of lactic acid bacteria harboring genes that promote the elimination of antinutritional factors

**DOI:** 10.3389/fpls.2023.1145041

**Published:** 2023-04-26

**Authors:** Hai-Ha-Thi Pham, Do-Hyung Kim, Thanh Luan Nguyen

**Affiliations:** ^1^ VK Tech Research Center, NTT Hi-Tech Institute, Nguyen Tat Thanh University, Ho Chi Minh City, Vietnam; ^2^ Department of Aquatic Life Medicine, College of Fisheries Sciences, Pukyong National University, Busan, Republic of Korea; ^3^ Department of Science and Technology, HUTECH University, Ho Chi Minh City, Vietnam

**Keywords:** LAB, α-GOS, tannin, saponin, phytates, lectin

## Abstract

Anti-nutritional factors (ANFs) substances in plant products, such as indigestible non-starchy polysaccharides (α-galactooligosaccharides, α-GOS), phytate, tannins, and alkaloids can impede the absorption of many critical nutrients and cause major physiological disorders. To enhance silage quality and its tolerance threshold for humans as well as other animals, ANFs must be reduced. This study aims to identify and compare the bacterial species/strains that are potential use for industrial fermentation and ANFs reduction. A pan-genome study of 351 bacterial genomes was performed, and binary data was processed to quantify the number of genes involved in the removal of ANFs. Among four pan-genomes analysis, all 37 tested *Bacillus subtilis* genomes had one phytate degradation gene, while 91 out of 150 Enterobacteriacae genomes harbor at least one genes (maximum three). Although, no gene encoding phytase detected in genomes of *Lactobacillus* and *Pediococcus* species, they have genes involving indirectly in metabolism of phytate-derivatives to produce Myo-inositol, an important compound in animal cells physiology. In contrast, genes related to production of lectin, tannase and saponin degrading enzyme did not include in genomes of *B. subtilis* and *Pediococcus *species. Our findings suggest a combination of bacterial species and/or unique strains in fermentation, for examples, two Lactobacillus strains (DSM 21115 and ATCC 14869) with *B. subtilis* SRCM103689, would maximize the efficiency in reducing the ANFs concentration. In conclusion, this study provides insights into bacterial genomes analysis for maximizing nutritional value in plant-based food. Further investigations of gene numbers and repertories correlated to metabolism of different ANFs will help clarifying the efficiency of time consuming and food qualities.

## Introduction

Plant-based protein originates from a variety of sources, the most common of which are soybean, sunflower, canola, and wheat. Plant-based proteins are typically characterized by an amino acid imbalance and high quantities of antinutritional factors (ANFs) such as indigestible non-starchy polysaccharides, phytic acid, tannins, and alkaloids, which limit their usage in animal nutrition formulations (see review by Popova and Mihaylova, 2019; Samtiya et al., 2020). Cereals and legumes are important staple foods in Asian nations due to their high quantities of macronutrients and micronutrients. Micronutrient availability in large quantities is not the only important nutritional component; bioavailability is also critical in meeting human dietary requirements. Micronutrient deficiencies, including those affecting vitamins and minerals, are thus one of the primary causes of metabolic health issues in low-income areas (Black et al., 2013).

Increased bioavailability of dietary components can improve a nutritional condition of community (Bouis et al., 2019). Due to their low cost and simplicity of growing, several plant-derived components (such as millets, paper mulberry, and alfalfa) have the potential to be used as an alternative feed source to reduce protein shortages and promote current livestock production growth. However, they are reported to have low nutritional value due to the presence of antinutritional agents that impair nutrient absorption (Sarita and Singh, 2016; Samtiya et al., 2020). For major staple foods that are consumed throughout the world, the cereals and legumes also contain high amounts of ANFs, which reduce the bioavailability of various components. Phytate, for example, is a primary antinutrient that chelates and predominantly impacts calcium and other minerals including iron, copper, and zinc bioavailability. Polyphenols and oxalates are two more antinutrients that can reduce dietary mineral bioavailability (Kaushik et al., 2018). Thus, several ANFs with hazardous potential have been tested in foods and proven to be heat stable or heat labile, and numerous traditional approaches and technologies aimed at lowering the amounts of these factors. Fermentation, germination, debranning, autoclaving, soaking, and other processes are used to minimize antinutrient levels in foods, such as the inactivation of soybean (trypsin inhibitors) and the use of phytases to deactivate phytates. It is possible to minimize the level of antinutrients in foods by utilizing numerous strategies alone or in combination.

The fermentation and storage procedure employing lactic acid bacteria (LAB) (also known as ensiling) was regarded to be the most efficient strategy for conserving nutrients and active chemicals in plant-based components and a novel way to deliver nutrition-rich green feed throughout non-growing periods (Chikwanha et al., 2018; Wang et al., 2021). In particular, Ensiling has also been used to lower the content of ANFs and other toxic or hazardous compounds in animal feed (Gefrom et al., 2013; Rinne et al., 2018). Thus, LAB inoculants have mostly focused on changes in nutritional value and active ingredient content after ensiling, with just a few reports of ANF decrease. As a result, the purpose of this study was to explore into a wide-genome selection of LABs that may maximize the impacts on lowering antinutritional content in plant-based materials such as legumes and cereals.

## Materials and methods

### Bacterial genome retrieved and pan-genome analysis

Comparative pan-genome analysis of hitherto-sequenced *Lactobacillus* spp., *Bacillus subtilis*, *Pediococcus* spp., and species of *Enterobacteriaceae* were performed with EDGAR v3.0 (Dieckmann et al., 2021). Accordingly, genomic subsets, including the number of core genome and singletons (strain-specific) in the gene pool, were extracted to understand the estimation of tracing horizontal gene flux across strains and obtain insights into their evolution.

### Functional analyses

For functional comparison, enzyme/compound involved in the metabolism of ANFs will be used to look into the functional gene within the pan-genome. The presence of gene related to each ANF metabolism in each genome will be retrieved and binary labeled 1. The total genes involved in each and all ANFs will be evaluated for each genome, and the total ANFs that the strain can metabolized was counted and compared to others strains either in the same family or not.

### Phylogenomic analyses

To determine phylogenomic relationships of species having higher gene numbers and ANFs, hierarchical clustering was performed based on numerous orthologous genes and comparative gene content, respectively. It is known that hierarchical clustering analysis of relatively shared gene content between genomes can display important differences in biological evolution and metabolic reconstructions (Sturn et al., 2002). Using EDGAR v3.0, both orthologous genes and relatively shared gene content between selected genomes were retrieved, and a phylogenetic tree was generated. A hierarchical clustering tree was constructed using Genesis v1.8.1 (Sturn et al., 2002).

## Results and discussion

### Phytate degradation by bacterial enzyme

Phytate (*myo*-inositol hexakisphosphate, IP6) may be found in a variety of ecosystems, including terrestrial and aquatic environments. This compound is produced by plants in the terrestrial ecosystem (Turner et al., 2002) and harbors a large portion of P under a complex calcium or magnesium salt, mostly in the seeds of most plants. Total P in cereal grains, oilseeds, and grain legumes is phytate P, with 1%–25% of total P found in diverse root and tuber forms and tiny quantities in leaves, while rice bran and other oilseed possess 56%–77% of P in total (Ravindran et al., 1994). Phytate has the ability to bind strongly to mineral surfaces, especially clays (Celi et al., 1999), and forms insoluble metal complexes (Dao, 2003; He et al., 2006). These characteristics may promote phytate resistant to microbial mineralization, resulting in its accumulation in soils (Turner et al., 2002). Multivalent positive cations such as Mg^2+^, Zn^2+^, Ca^2+^, Fe^2+^, and Mn^2+^, and amino group derivatives in protein moieties can be chelated by phytic acid, resulting in lower nutritional solubility, bioavailability, and absorption in animals (Waters et al., 2015).

Plants, animals, and microbes are all known to produce phytases (EC 3.1.3.8, 3.1.3.26, and 3.1.3.72) (Konietzny and Greiner, 2002). Following the hydrolysis of the phytate, solubilized forms of inorganic phosphate, inositol phosphate, and *myo*-inositol are released (Askelson et al., 2014; Zeller et al., 2015). Phytases are categorized as 3-phytases or 6-phytases based on the location of the phosphate that is hydrolyzed first (da Silva et al., 2019).

Fermented foods offer a variety of functional properties that provide consumers with multiple benefits (for example, antioxidation, digestive enzyme, probiotic potentials, antibacterial activities, bioactive peptides, etc.) (Tamang et al., 2016). In addition, fermenting microorganisms can produce a range of enzymes (e.g., phytase, protease, lipase, amylase, etc.) that hydrolyze lipids, carbohydrates, and proteins into simple digestible components with desirable texture and flavor. In particular, microbial enzymes may also break down antinutritional substances such as protease inhibitors, tannins, and phytates. Although humans lack endogenous phytases and do not have a microbial community capable of producing them in their small intestine (Lei et al., 2007), phytate-hydrolyzing lactic acid bacteria (LAB-Pht+) have been widely used in phytate-rich food processing. Under solubilized forms of phytate, different metal ions may be generated, increasing their bioavailability up to severalfold (Gupta et al., 2015), thus facilitating uptake and absorption. In addition, as a by-product of phytate hydrolysis, multiple *myo*-inositol(s) are produced, which play an important role in the control and regulation of several metabolic processes. This has increased interest in using fermentation to improve nutrient bioavailability and absorption in plant seeds (Dhull et al., 2020). However, employing microbial enzymes as human food additives is still a risk (Brodmann et al., 2017). Attempts were made to isolate microorganisms capable of producing phytase while being safe for humans (Sun et al., 2019). Endogenous enzymes are active during food fermentation as a result of reduced pH, which contributes to phytic acid reduction.

**Figure 1:**
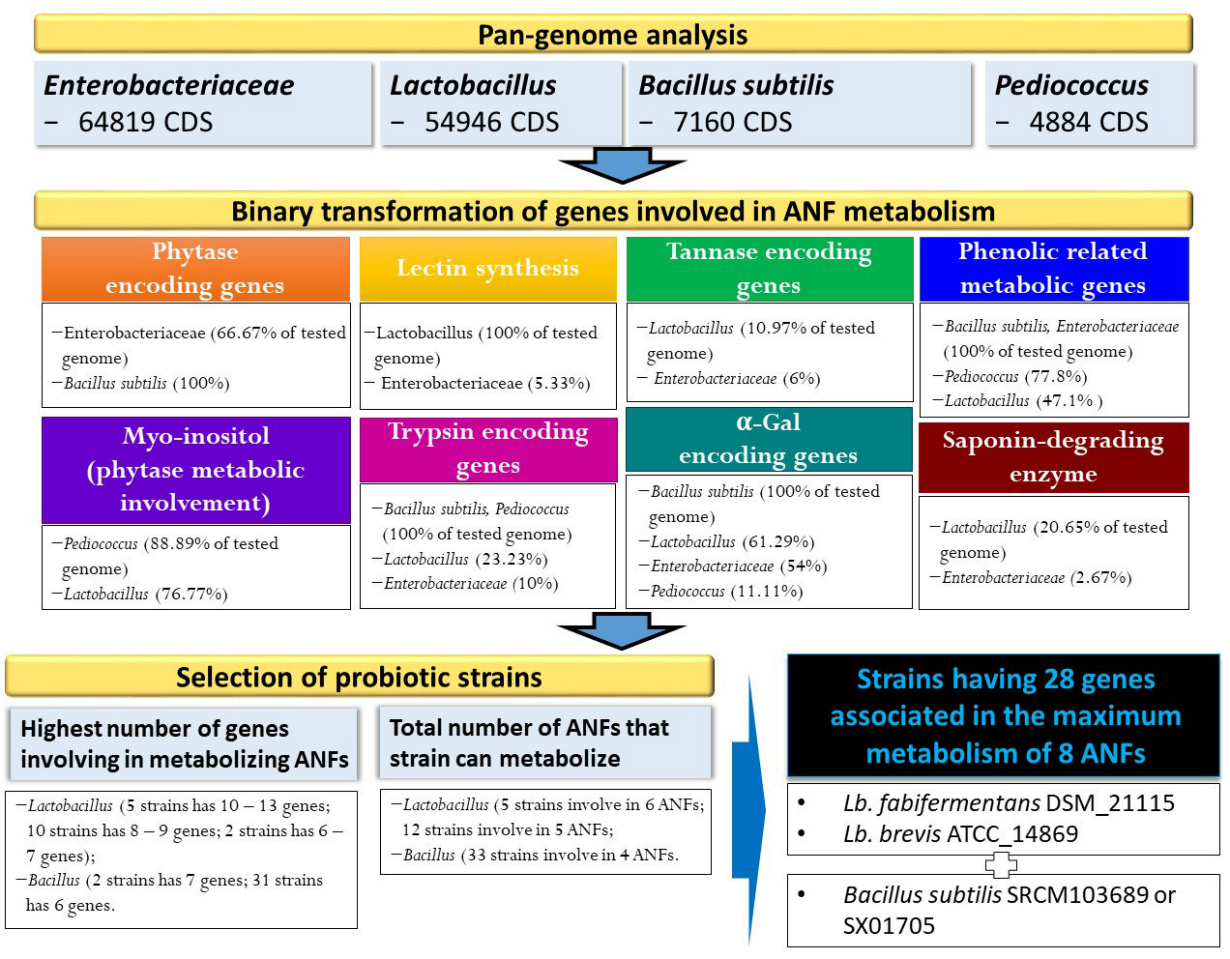
Identification of ANFs encoding genes based on pangenome analysis and strain selection based on binary data conversion.

**Figure 2:**
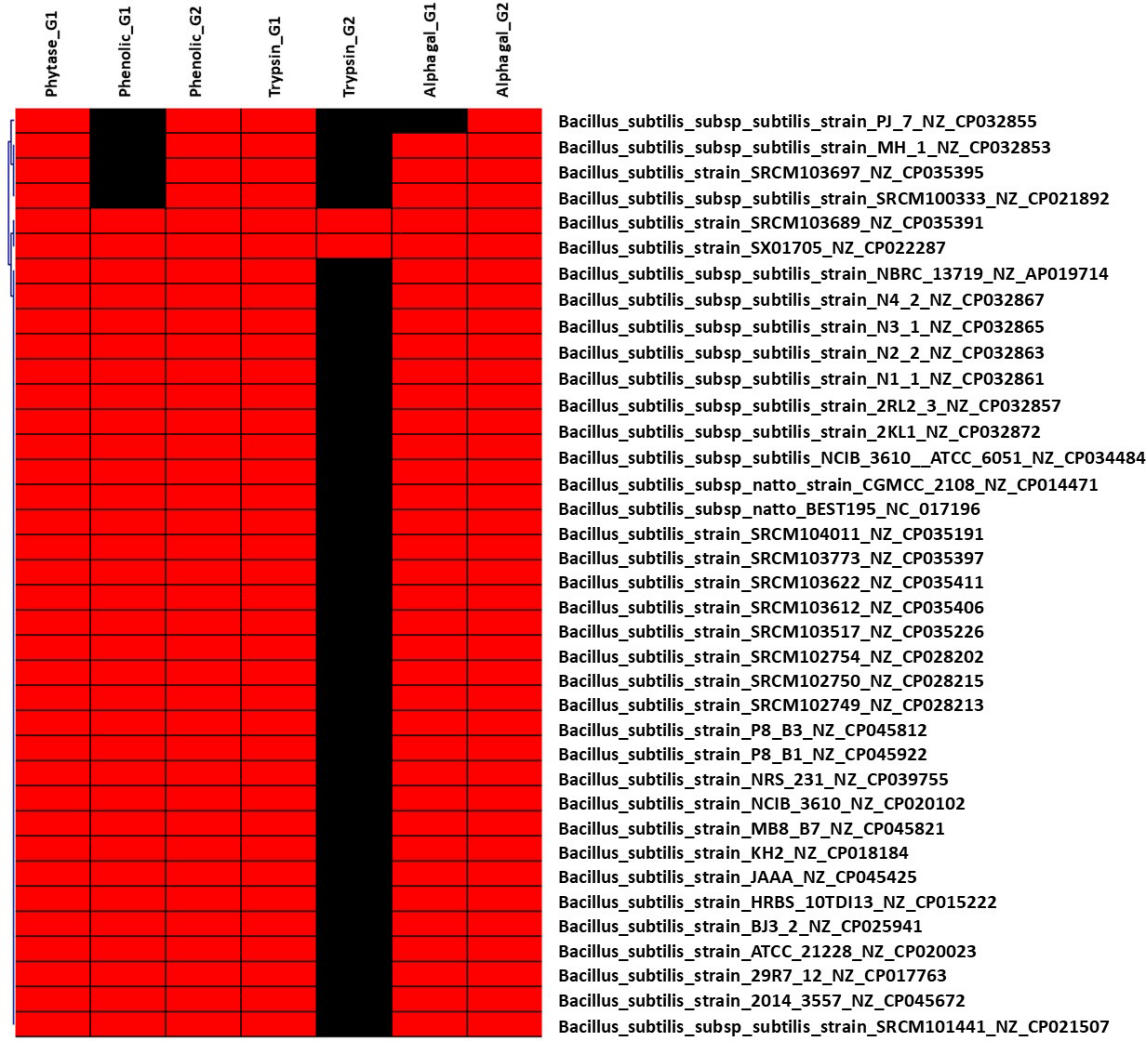
Hierarchical clustering of dispensable genes potentially involved in the metabolism of ANFs of *B*. *subtilis*. The accession number of genes were detailed in **Supplementary Table S1**. The presence and absence of genes in each genome are indicated in red and black, respectively. Each strain is included with genome accession number.

Various microorganism including bacteria, yeasts, and filamentous fungus (Pandey et al., 2001; Reale et al., 2004; Jorquera et al., 2008; Javed et al., 2010; Wyss et al., 1999) have been discovered as phytase producers. However, not all strain can produce phytase, thus screening for strain harboring the gene encoding for phytase enzyme will be necessary. Indeed, based on such pan-genome distribution, 91 out of 150 Enterobacteriaceae genomes (60.67%) harbor gene(s) encoding for phytase enzymes (**Supplementary Table S1**), as shown in **Figure 1**. Most of genomes (60/92 genomes) followed by 28 genomes harbor one and two genes encoding for phytase, respectively. In particular, three genomes were discovered with three phytase-encoding genes, which are species of *Edwardsiella ictaluri* ATCC 33202 and *E. tarda* ATCC_15947 and ET883. On the other hand, pan-genome analysis of 37 *B. subtilis* strains showed that all strains contain gene encoding for phytase enzyme (**Figure 2**). Our findings support that of the previous study that some bacterial species such as *Bacillus* and family of Enterobacteriaceae such as *Citrobacter*, *Escherichia*, *Enterobacter*, *Klebsiella*, and *Pseudomonas* has been reported to harbor phytase activity and produce inositol (Justin et al., 2003; Jorquera et al., 2008; Yamaoka et al., 2011).

### Phytate degradation and its by-product *Myo*-inositol functional role in health

*Myo*-inositol (MI) is produced by gastrointestinal phytate breakdown from plant sources, and it has acquired importance in animal cells physiology (Holub, 1986; Selle and Ravindran, 2007; Kim et al., 2017). For example, MI has been shown to be essential in a variety of metabolic and regulatory activities. This is a component in vitamin B group involved in lipid signaling, osmolality, glucose, and insulin metabolism. However, this notion was disproved because monogastric animals and humans could depend on cellular biosynthesis (Regidor and Schindler, 2016). Body-own synthesis satisfied most of the MI requirements for human newborns (Brown et al., 2009). Dietary MI has been demonstrated to be useful in treating some endocrine diseases such as diabetes and glucose intolerance (Croze and Soulage, 2013). Thus, MI may be considered a semiessential substance that may be limited under certain physiological and pathological conditions. Then, supplementation of gut bacteria involved in the metabolism of inositol will help to retain its important biological functions.

**Figure 3:**
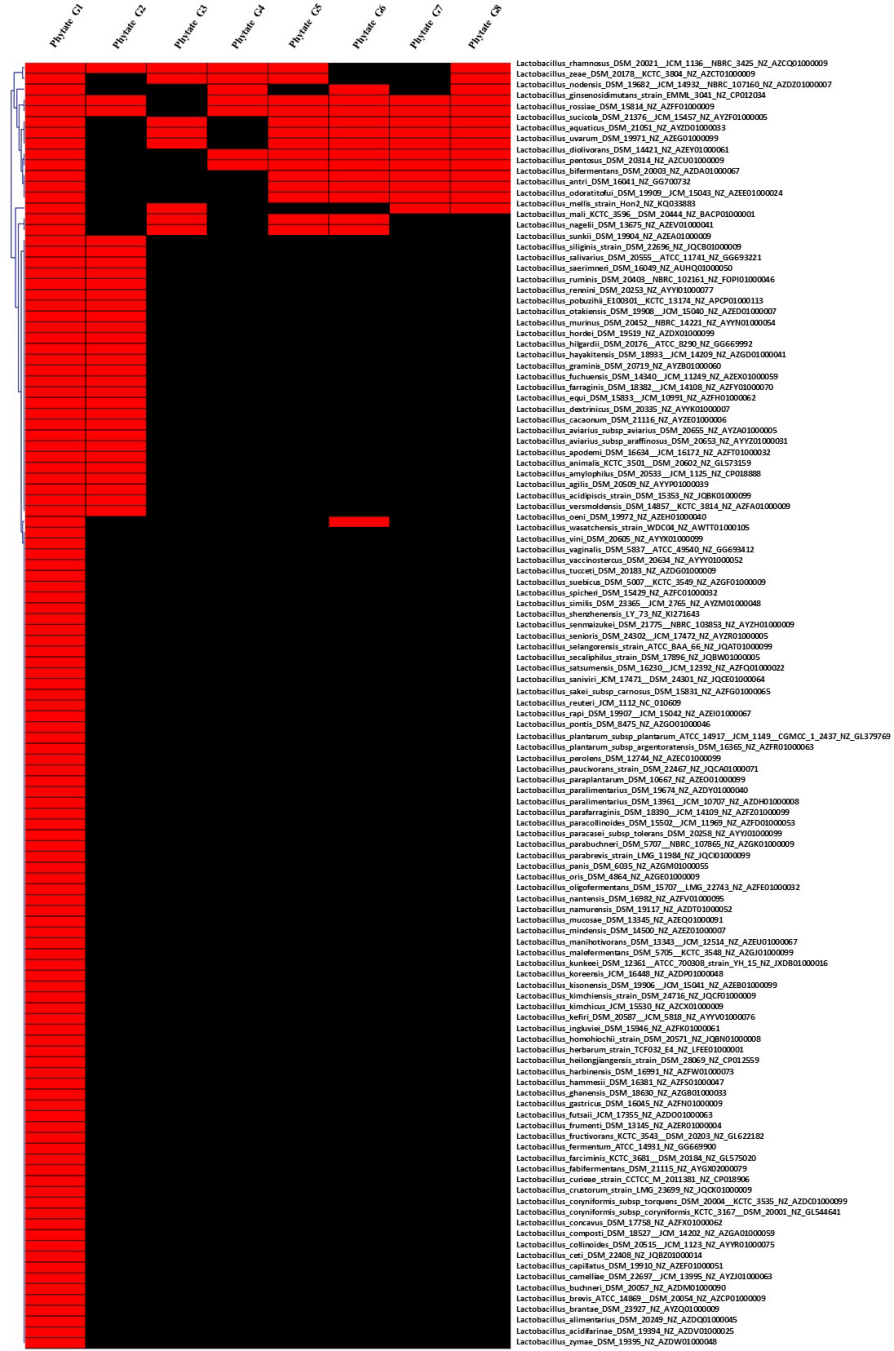
Hierarchical clustering of dispensable genes potentially involved in phytate metabolism of species belonging to Lactobacillaceae. The accession number of genes are detailed in **Supplementary Table S2**. The presence and absence of genes in each genome are indicated in red and black, respectively. Each strain is included with genome accession number.

In this study, the results of pan-genome analysis of Lactobacillaceae showed that 119 of 155 genomes contained at least one gene encoding for phytate metabolism (**Supplementary Table S2**). Most of the tested strains (76/119 genomes) harbor only one gene involved in phytate metabolism, followed by 27 genomes harboring two genes, four genomes harboring four and five genes. In particular, six genomes harboring six genes involved indirectly in phytate metabolism (**Figure 3** and **Supplementary Table S2**) are *Lactobacillus diolivorans* DSM_14421 (NZ_AZEY01000061), *Lactobacillus pentosus* DSM_20314 (NZ_AZCU01000009), *Lactobacillus rhamnosus* DSM_20021 (NZ_AZCQ01000009), *Lactobacillus aquaticus* DSM_21051 (NZ_AYZD01000033), *Lactobacillus sucicola* DSM_21376 (NZ_AYZF01000005), and *Lactobacillus uvarum* DSM_19971 (NZ_AZEG01000099), and two genomes harboring seven genes involved in phytate metabolism are *Lactobacillus ginsenosidimutans* strain_EMML_3041 (NZ_CP012034) and *Lactobacillus rossiae* DSM_15814 (NZ_AZFF01000009). Thus, we hypothesize that the combination of *Lactobacilus* and *Bacillus* should be evaluated in future studies on the metabolism of phytate and generation of the beneficial compound of MI.

### Tannin degradation by bacterial enzyme

Tannin is a significant antioxidant polyphenol with multifunctional characteristics for human health. These oligomers of flavan-3-ols and flavan-3, 4-diols are mostly found in the bran fraction of legumes (Ngozi, 2014). In addition, this water-soluble polyphenol is found in grapes and green tea (Chu et al., 2015). However, some studies have determined that goats are resistant to these tannins, but cattle and sheep are sensitive (D’Mello, 2000; Bhattarai et al., 2016; Smeriglio et al., 2017). When tannins are consumed, they form compounds with proteins, which induce the deactivation of numerous digestive enzymes and a reduction in protein digestibility (Joye, 2019). Thus, an approach of fermentation removing the tannin is ideal to improve the workload in the digestive system.

Our analysis of genomes retrieved from Enterobacteriaceae showed that nine species harbor gene(s) encoding for tannase (**Supplementary Table S1**). Among species belonging to Enterobacteriaceae, *Rouxiella chamberiensis* strain 130333 (NZ_JRWU01000102) and *Lonsdalea quercina* strain ATCC_29281 (NZ_FNQS01000022), which were isolated from parenteral nutrition bags for newborns (Le Flèche-Matéos et al., 2015) and oak trees showing symptoms of drippy blight (Ibarra Caballero et al., 2014), respectively, contain three and two genes related to tannase, respectively. In addition, this study shows a *Rahnella aquatilis* CIP 78.65 plasmid (NC 016818 and gene RAHAQ2 RS23995) recovered from a drinking water source in Lille, France (Martinez et al., 2012) has a gene encoding for tannase (**Supplementary Table S1**). In particular, *Rahnella* members have been studied for their possible use in plant growth and nutrient acquisition, food sciences, and bioremediation (e.g., Kim et al., 1997; Kang et al., 2004; Kourtev et al., 2006). Thus, our findings show that tannese-harboring species may be involved in degrading numerous ANFs from plant components for nutrients. However, when choosing bacteria from the Enterobacteriaceae family for use in plant-based feed fermentation, the origin of the isolates, and their pathogenicity potential, must be examined.

**Figure 4:**
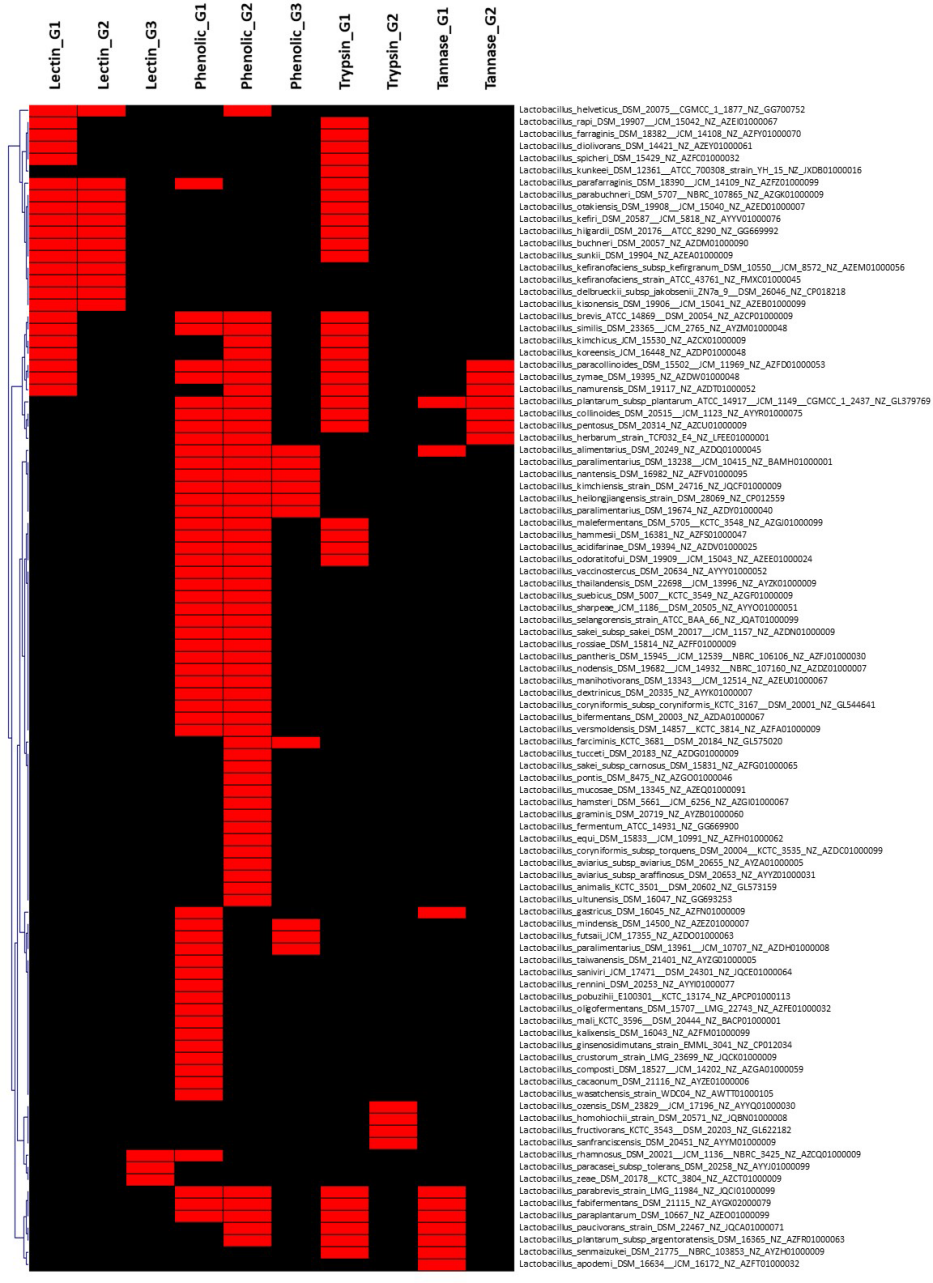
Hierarchical clustering of dispensable genes potentially involved in the metabolism of lectin, phenolic compounds, and tannin of species belonging to Lactobacillaceae. The accession number of genes are detailed in **Supplementary Table S2**. The presence and absence of genes in each genome are indicated in red and black, respectively. Each strain is included with genome accession number.

On the other hand, the pan-genome analysis of Lactobacillaceae resulted in 17 genomes (species) out of 155 genomes, which harbor a gene encoding for tannase (**Figure 4** and **Supplementary Table S2**) with the exception of *Lactobacillus plantarum* subsp. *plantarum* ATCC_14917 that harbors two genes related to protein encoding tannase. These findings suggest that screening for tannase-coding genes in the genomes of *Lactobacillus* species is required before strains may be expected to remove tannin from plant products during fermentation. This is consistent with previous research that found that lactic acid bacteria (*L. plantarum*) may considerably enhance the fermentation quality of paper mulberry and minimize the quantity of antinutrient components such as tannin (Wang et al., 2022).

### Metabolism of phenolic compound by bacterial enzyme

Phenolic compounds are plant secondary metabolites that include one or more hydroxyl groups on the aromatic ring. Phenolic acids, a subclass of plant phenolics, have a phenol moiety and a resonance stabilized structure, which results in H-atom donation and antioxidant activity *via* a radical scavenging mechanism. These key components participate in the formation of molecular linkages in plant cell walls between cellulose, hemicellulose, and lignin (Pei et al., 2020). They are frequently studied because their unusual structure endows phenolic acids with critical biological activities (Shahidi and Peng, 2018), such as the capacity to scavenge free radicals, giving them possible health advantages (antioxidant, anti-inflammatory, antimicrobial, and cardioprotective characteristics). In addition, they are easily absorbed in the gut, making them suitable for use as bioactive ingredients in the formulation of dietary supplements.

On the other hands, phenolic chemicals have been extensively researched for their potential environmental toxicity and hazards to human health (Chen et al., 2010). Because some bioactive polyphenolic compounds have varying effects on metal chelation, their presence in meals enhances astringency and may inhibit the absorption of certain minerals such as iron and zinc. Heat processing can cause phenolic molecules to oxidize, and the resultant oxidized phenolics, such as quinones, can interact with amino acids, leaving them nutritionally inactive (see review by Shahidi, 1997). It is prudent to study if regular eating of bioactive polyphenolic components impairs iron use (Kim et al., 2008). Gossypol, a polyphenolic compound found in African cotton seed (*Gossypium* spp.), has been documented to be very toxic to pigs, causing dyspnea, anorexia, unthriftiness, and diarrhea (Chen et al., 2010). Some phenolics are especially toxic to aquatic organisms such as fish and shellfish at extremely low quantities (Guerra, 2001; Enguita and Leitão, 2013).

Phenolic substances widely generated in plants (e.g., catechol and hydroquinone) may be cell transformed and are genotoxic to various species, including microbes and human cells (Chen et al., 2010). Fungi and bacteria may both biodegrade phenolic substances. Some bacteria contain a detoxifying system mediated by the phenolic acid decarboxylase (PAD) enzyme, which may synthesize phenolic acid, release carbon dioxide, and create less toxic vinyl derivatives (Natella et al., 1999). PADs may have potential uses as biocatalysts, since vinyl derivatives of phenolic acids may be employed as polymer precursors and are also of interest in the food-processing sector.

In pan-genome analysis of strains in Enterobacteriaceae, gene-encoding proteins involved in metabolism of phenolic substance including glutathione-dependent reductase, 3-polyprenyl-4-hydroxybenzoate carboxy-lyase (*Ubi*X), hydroxyaromatic non-oxidative decarboxylase C, phenolic acid decarboxylase, and phenolic acid decarboxylase subunit B were found in genomes. Among the strains, 32 out of 150 genomes contain at least five genes, and two genomes have six genes in species *Enterobacter soli* ATCC BAA_2102 (NZ_LXES01000074) and *Kluyvera ascorbata* ATCC 33433 (NZ_JMPL01000253) (**Supplementary Table S1**). In general, all tested genomes harbor genes related to 3-polyprenyl-4-hydroxybenzoate carboxy-lyase (UbiX), indicating its roles in biosynthesis in ubiquinone and other terpenoid-quinone through the catalysis of substrates with different polyprenyl tail lengths to 2-polyprenyl phenol. PAD enzymes catalyze the conversion of phenolic acid derivatives such as ferulic or p-coumaric acids into the corresponding volatile molecules 4-vinyl guaiacol (3-methoxy-4-hydroxystyrene) or 4-vinyl phenol (4-hydroxystyrene), which are assumed to be vanillin precursors (4-hydroxy-3-methoxybenzaldehyde) (Koseki et al., 1996; Landete et al., 2010).

In addition, characteristics of bacterial PAD have been reported in *L. plantarum* (Cavin et al., 1997; Rodríguez et al., 2008), *Pediococcus pentosaceus* (Barthelmebs et al., 2000), *B. subtilis* (Cavin et al., 1998), and *B. pumilus* (Zago et al., 1995). In line with these findings, pan-genome analysis showed that 73 out of 150 genomes of *Lactobacillus* species harbor gene(s) encoding for PAD enzyme, indicating that not all of *Lactobacillus* strains involved in the metabolism of phenolic acid. In particular, only six genomes of five species (**Supplementary Table S2**) including *Lactobacillus alimentarius* DSM_20249 (NZ_AZDQ01000045), *Lactobacillus heilongjiangensis* DSM_28069 (NZ_CP012559), *Lactobacillus kimchiensis* DSM_24716 (NZ_JQCF01000009), *Lactobacillus nantensis* DSM_16982 (NZ_AZFV01000095), and *Lactobacillus paralimentarius* DSM_13238 (NZ_BAMH01000001) consist of three genes encoding for PAD enzyme. Only one and two genes for PAD enzyme were found in the genome of *L. plantarum* subsp. *argentoratensis* DSM_16365 (NZ_AZFR01000063) and *L. plantarum* subsp. *plantarum* ATCC_14917 (NZ_GL379769), respectively. In contrast, all genomes of *Bacillus* species tested include maximum two genes coding for PAD, while seven out of nine genomes of *Pediococcus* harbor only a gene. These results indicated that *Lactobacillus* strains might be a good selection for working as a source of phenolic compound elimination compared to *Bacillus* and *Pediococcus*. We also considered that genome comparison plays important roles in the selection of probiotic strain including its functions in eliminating phenolic compounds that might be a unique characteristic of bacteria adapting to their microenvironment (Nguyen and Kim, 2018). Although the number of genes revealed in these genomes differs, the molecular characteristics and biochemical capability for phenolic acid metabolism, whether dependent on the number of genes discovered in the same bacterial species or not, must be explored.

### Inhibition of lectin binding and production of probiotic lectin

In general, cereals, legumes, and tubers have large amounts of lectin, which are glycoproteins that easily attach to red blood cells and cause agglutination. The ingestion of lectin-containing meals would impair the transport and hydrolytic activities of enterocyte (Krupa, 2008). Natural lectins (e.g., uncooked or partially cooked kidney beans) interact with carbohydrates on the surface of epithelial cells. The attachment of lectins results in morphological and physiological alterations, such as damage to the luminal membranes of the epithelium, interference with nutritional digestion and absorption, and changes in the gut microflora (Banwell et al., 1993; Vasconcelos and Oliveira, 2004; Hamid et al., 2013). As a result of these “bad lectin” attachments to intestinal epithelial cells, systemic nutrition homeostasis is disrupted, internal organ and tissue growth and/or atrophy occurs, and hormonal and immunological function is altered (Vasconcelos and Oliveira, 2004).

On the other hand, lectin damages the digestive tract, enabling opportunistic bacteria into the circulation (Muramoto, 2017). It is stable in a wide pH range and resistant to proteolytic enzymes, allowing it to transit through the gastrointestinal system to interact with carbohydrates on the surface of plant-eating animal epithelial cells. Thus, the deleterious effects of lectins are dependent on their ability to withstand degradation in the intestine and their specificity to membrane polysaccharides moieties produced by epithelial cells. The “bad lectin” can disrupt digestive enzymes, cause brush border membrane shedding, and shrink microvilli, all of which contribute to a decrease in absorptive function and nutrition utilization while increasing cellular proliferation and turnover.

Probiotic bacteria supplementation has been proposed to help in the binding and elimination of certain lectins and to prevent intestinal epithelial damage (Zárate and Perez Chaia, 2009). Obviously, the probiotic-modifying composition of the gut microbiota may have a role in the binding and removal of lectins contained in food, although this has not been clinically investigated. This study suggests that probiotic cells with high adhesion abilities help in the maintenance of the gut barrier and metabolic processes. It necessitates not only the development of digestive enzymes for the breakdown of “bad lectins” (Peumans and Van Damme, 1996) but also the attachment of lectin to host cells.

Moreover, the term “probiotic lectins” has been mentioned in relation to their systems and lectin ensembles (Lakhtin et al., 2007). Individual lectins may display beneficial behavior such as switching on/off metabolic cascades and enhancing compatibility and biodegradability (producing signals) in nearby microbial and host systems. Pidgorskyi et al. (2017) observed that *Bacilli* lectins efficiently blocked surface sialic acid-containing receptors of several pathogenic agents (e.g., influenza, herpes, hepatitis C, and HIV), restricting not only their adsorption and reproduction but also the appearance and progression of viral infection. Previous research (Lakhtin et al., 2006; Lakhtin et al., 2007) demonstrated that isolated lectins from probiotic bacteria (e.g., *Lactobacilli* and *Bifidobacteria*) play a variety of key biological activities, including cell-surface-building components, cytokines, biofilm organization, co-function with metal cations, and hydrolases (see review by Lakhtin et al., 2007).

In this study, 26 out of 150 genomes were found to have lectin-encoded gene(s) with three different gene structures. Among them, 12 genomes have similar two gene structures encoding for different lectins, while 14 genomes harbor only one gene encoding for two different structures of lectin, which can be divided into two sub-groups including 11 genomes and three other genomes (**Supplementary Table 2**). However, only 8 (out of 150) genomes of Enterobacteriaceae harbored gene(s) encoding five different structures of lectin (**Supplementary Table S1**), while no gene encoding for lectin was observed in genomes of *B. subtilis*. Our findings support previous studies that suggest that lectin structural diversity is due to environmental adaptation (Lakhtin et al., 2007; Pidgorskyi et al., 2017). Acidic probiotic lectins may have higher immunomodulatory (immunogenic) effects, whereas basic lectins may have enhanced antibacterial and antiviral characteristics (detergent-like) (Lakhtin et al., 2007). In general, acid and basic lectins derived from the same source can work together to provide the more significant impacts expected for probiotics.

### Trypsin enzyme

Trypsin (EC 3.4.21.4) is a crucial enzyme in the digestive tract of many vertebrates that is generated by the pancreas and released into the duodenum. It may hydrolyze proteins into peptides and activate chymotrypsin zymogens and other pancreatic enzymes. Trypsin inhibitors, on the other hand, cause trypsin and chymotrypsin to be lost in the intestines, reducing its ability to hydrolyze proteins into peptides during the foodstuff digestion process. Grain legumes (e.g., chickpeas, soybeans, red kidney beans, and other Leguminosae, Solanaceae, and Gramineae members) provide 10% of worldwide dietary protein and include various ANFs, including trypsin inhibitors (see review by Popova and Mihaylova, 2019). For example, the Kunitz type (>20 kDa) is a major protease inhibitor found in significantly greater concentrations in soybeans, and it works by forming stable stoichiometric interactions with the digesting enzymes trypsin and chymotrypsin (Liener, 1994). These non-covalent compounds render proteases inactive and significantly reduce non-ruminant protein and amino acid (AA) digestion and utilization (Rawlings et al., 2004).

Exogenous protease supplementation is alternatively being used to boost trypsin activity, reduce the adverse effects of trypsin inhibitors, and improve the digestibility of plant-based products (e.g., Erdaw et al., 2017; Aderibigbe et al., 2020; Asare et al., 2022). In addition, the fungal and bacterial including lactic acid bacteria (LAB) protease were discovered to be able to destroy or inactivate the trypsin inhibitor (by outcompeting for active sites) in raw plant materials *in vivo* (Huo et al., 1993; Mustafa et al., 2020). Hence, screening for exogenous trypsin secretion by using bacterial genomes should be included in the criteria selection of probiotic bacteria applied for the animal species (e.g., birds, non-ruminants) that may be impaired when ingesting antinutrients in feed ingredients and/or for specific species where insufficient secretion of proteolytic enzymes are required for digestion (Asare et al., 2022).

Although several trypsins and trypsin-like proteases (TLPs) may be manufactured by microorganisms such as *Trichoderma* and *Bacillus*, this may have a number of risk for use in therapeutic drugs. TLP enzymes can execute the same function as trypsin and can be synthesized by LAB (e.g., Wulansari et al., 2012; Margono et al., 2014). In this study, 15 out of 150 genomes of Enterobacteriaceae have only one (in total three different genes) gene encoding for trypsin; 36 out of 155 genomes of Lactobacillaceae harbor one gene are involved in the synthesis of trypsin-like serine protease with two different genes (**Supplementary Table S2**). In particular, genes encoding for trypsin were found in all genomes of *B. subtilis* (**Supplementary Table S3**) and *Pediococcus* (**Supplementary Table S4**).

Dispensable genes expressing trypsin or TLP enzymes may demonstrate different proteolytic activity in response to diet. For example, homologous genes encoding for TLP enzyme in the genome of *L. plantarum* subsp*. plantarum* ATCC 14917 (NZ_GL379769) was shared to other 31 *Lactobacillus* genomes (**Supplementary Table 2**), whereas four genomes including *L. fructivorans* KCTC 3543 (NZ_GL622182), *L. homohiochii* DSM 20571 (NZ_JQBN01000008), *L. ozensis* DSM 23829 (NZ_AYYQ01000030), and *L. sanfranciscensis* DSM 20451 (NZ_AYYM01000009) shared different genes encoding for TLP enzyme. This finding is consistent with a previous study that showed that the secreted TLP1 of *L. plantarum* subsp*. plantarum* PTCC 1896 might hydrolyze the trypsin-specific substrate (Mustafa et al., 2020). Thus, essential molecular screening for probiotic strain selection would be enhanced by including the gene encoding for trypsin and/or TLP enzyme. Two genes encoding trypsin were identified in the genomes of *B. subtilis* SRCM103689 (NZ CP035391) isolated from food and SX01705 (NZ CP022287) isolated from mushroom substrate. This finding suggests that these two bacterial strains have the ability to produce trypsin, which is being researched for usage in the feed industry to replace expensive exogenous enzymes (Asare et al., 2022).

### Degradation of saponin by bacterial enzyme

Saponins are secondary plant compounds found in legumes, sunflower seeds, spinach leaves, tea leaves, quinoa seeds, sugar beets, and allium species. Due to the inhibitory effects of digestive enzymes such as amylase, glucosidase, trypsin, chymotrypsin, and lipase, these antinutrients might induce indigestion-related health problems (Liener, 2003; Ali et al., 2006; Birari and Bhutani, 2007; Lee et al., 2015; Ercan and El, 2016). In particular, saponins contain amphipathic molecules with hydrophilic sugar moieties and a hydrophobic pentacyclic triterpene backbone, making it critical for them to replace cholesterol in the cytomembrane (Vo et al., 2017). Thus, these saponin-containing substances are undesired hemolytic components that must be destroyed in order to be employed in animal feed.

Saponin structures in tea seed were recognized by the involvement of polar groups of the aglycone and the amount of sugar units (tetrose) linked by a β1→4 glycosidic bond (Morikawa et al., 2007; Myose et al., 2012). It was predicted that β-glucuronidase would break these glycosidic bonds in saponins, but as this enzyme does not exist in the human digestive system, gastrointestinal digestion may be significantly impeded (Amin et al., 2011). In addition, such bacteria and fungi (e.g., *Streptococcus* spp., *Bacillus* spp., *Stagonospora >avenae*, and *Aspergillus niger*) synthesize saponin-degrading enzymes and facilitate modification into less hazardous saponins with poor cytolytic activity (Garcia-Amado et al., 2007; Wie et al., 2007). In a previous study (Qian et al., 2018), *Lactobacillus crustorum* isolated from raw bovine milk can produce endo-β-glucuronidase that hydrolyze saponins in the tea seed and might help to reduce its hemolytic activity *in vitro*. This suggests that screening for gene encoding β-glucuronidase should be involved in a multi-omics selection of saponin-degrading microbial strains.

In this study, one and five genes encoding β-glucuronidase was extracted from gene repertories of tested Enterobacteriaceae and *Lactobacillus* genomes, respectively, but it is not present in genomes of *Pediococcus* and *B. subtilis*. Among 151 genomes, 31 genomes of *Lactobacillus* species harboring at least one gene encoding β-glucuronidase with maximal numbers of genes (four genes) were predicted for *L. hammesii* DSM_16381 (NZ_AZFS01000047), *L. parabrevis* strain LMG_11984 (NZ_JQCI01000099), and *L. secaliphilus* strain DSM_17896 (NZ_JQBW01000005). Of 150 genomes, *Escherichia coli* DSM_30083 (NZ_KK583188), *Edwardsiella ictaluri* ATCC_33202 (NZ_AFJI01000117), *Edwardsiella tarda* ATCC_15947 (NZ_BANW01000001), and *Pantoea ananatis* LMG_2665 (NZ_JFZU01000028) are unique strains harboring gene encoding β-glucuronidase in genome (**Supplementary Table S1**). These findings indicate that the most species appropriate for the reduction in hemolytic activity are *Lactobacillus*, but it needs to be investigated whether numerous genes encoding β-glucuronidase will withdraw quickly the saponins or degrade varied saponins from other components.

### Degradation of α-GOS by bacterial enzyme

Low concentrations of antinutrients such as phytic acid, lectins, phenolic compounds, protease inhibitors, and saponins have been shown to lower blood glucose and/or plasma cholesterol and triacylglycerols (Shahidi, 1997). Several enzyme inhibitors found in nearly all cereals and legume-based diets, especially, inhibit α-amylase activity. These inhibitors increase carbohydrate digestion time and decrease glucose absorption rate, altering glucose levels in healthy postprandial plasma (Bhutkar and Bhise, 2012). In contrast, legume grains are also known to be a rich storage of carbohydrates that protects its physiological functions, but it is a non-digestible ingredient in the mammalian digestive tract. It is due to that fact that their structures are characterized as α (1 → 6) linked galactosyl derivates from sucrose, called α-galactosides (α-GOS), and the mammalian upper gastrointestinal tract lacks the enzyme, galactosidase, resulting in the poor hydrolytic digestion of α-GOS. These non-digestible oligosaccharides are rapidly digested by indigenous bacteria, resulting in the generation of huge volumes of gases in the colon. This induced flatulence significantly reduces the attractiveness of soy products as a primary food source for human and animals.

The utilization of microbial α-Gal is a potential strategy for the degradation of these undesirable α-GOS and the synthesis of good short-chain fatty acids. In this study, genes encoding for α-Gal were targeted to identify bacteria that promote α-GOS fermentation. As a result, six different genes encoding for α-Gal were found in 81 of 150 Enterobacteriaceae genomes, with 15 genomes having two genes and two genomes having three genes (**Supplementary Table S1**). As a result, six distinct genes encoding for α-Gal were found in 81 of 150 Enterobacteriaceae genomes, with 15 genomes including two dispensable genes and two genomes containing three dispensable genes (**Supplementary Table S1**). All *B. subtilis* genomes include two genes encoding for α-Gal, with the exception of *B. subtilis* subsp. *subtilis* strain PJ_7 (NZ CP032855) (one gene). Two α-Gal-coding genes were found exclusively in the genome of *P. pentosaceus* strain DSM 20336 (NZ JQBF01000009). In particular, 29 dispensable genes were distinguished in 95 (out of 155) genomes of Lactobacillaceae (**Supplementary Table 2**), indicating its diversity in the metabolism of α-GOS that may link to derivates from sucrose with the most common homologues raffinose, stachyose, and verbascose. *Lactobacillus rhamnosus* DSM_20021 (NZ_AZCQ01000009) is the strain harboring the highest number of genes encoding for α-Gal (10 genes) followed by *L. shenzhenensis* LY_73 (NZ_KI271643) and *L. perolens* DSM_12744 (NZ_AZEC01000099) (eight dispensable genes), *L. zeae* DSM_20178 (NZ_AZCT01000009) and *L. paralimentarius* DSM_19674 (NZ_AZDY01000040) (seven dispensable genes), and *L. paralimentarius* DSM_13961 (NZ_AZDH01000008), *L. kisonensis* DSM_19906 (NZ_AZEB01000099), and *L. ultunensis* DSM_16047 (NZ_GG693253) (six dispensable genes).

### Selection of probiotic strains with maximal capability to reduce ANFs

Microbial feed components or probiotic supplements have been considered for increasing production efficiency. They must be derived from their hosts to be resistant to extreme temperatures, acidic pH, and desiccation. To uncover required probiotic characteristics, a screening approach and comprehensive multi-omics characterization were recently used. However, these strategies may only be aimed at preventing probiotic strain misidentification in product labeling and anticipating the hazard of strains carrying virulence factors, toxins, antibiotic resistance, or toxic metabolites. In particular, our previous study (Nguyen and Kim, 2018) showed that not all GRAS strains can be targeted as probiotics, and comparative genomics techniques are highly potent tools for predicting probiotic characteristics. In addition, fermentation is considered as alternatives to reduce such antinutrient types in plant-based product (Qian et al., 2018; Popova and Mihaylova, 2019; Samtiya et al., 2020), but time consumption and efficiencies are strain dependent.

In this study, the number of genes involved in the process of antinutrient metabolism and types of ANFs that can be neutralized by different bacterial genomes/strains were predicted. Among four pan-genomes analyses for its participation in phytate degradation, such species that belonged to the family Enterobacteriaceae (91/150 genomes) and *B. subtilis* (all tested genomes) can degrade phytate directly by the production of phytase, whereas species that belonged to *Lactobacillus* and *Pediococcus* may be involved indirectly in the metabolism of phytate to produce *Myo*-inositol. In contrast, genes related to the production of lectin-, tannase-, and saponin-degrading enzymes were not in the genomes of *B. subtilis* and *Pediococcus* species, indicating that these metabolisms maybe unique in specific species/family, which symbiotically support other microorganisms under same environments of diverse nutrients. Interestingly, such pathogenic species belonging to Enterobacteriaceae have genes encoding for multi-functions in reducing seven ANFs including phytase, tannase, lectin, phenolic, trypsin, α-Gal, and/or saponin-degrading enzymes with maximum metabolism of four ANFs belonging to 12 genomes including *K. quasipneumoniae* subsp. *quasipneumoniae* 01A030 (NZ_CCDF01000065), *Cronobacter malonaticus* LMG_23826 (NZ_CP013940), *Cronobacter sakazakii* ATCC 29544 (NZ_CP011047), *Enterobacter ludwigii* EN_119 (NZ_CP017279), *K. quasipneumoniae* subsp. *Similipneumoniae* 07A044 (NZ_CBZR010000046), *K. variicola* DSM_15968 (NZ_CP010523), *Franconibacter helveticus* LMG 23732 (NZ_AWFX01000057), *P. ananatis* LMG 2665 (NZ_JFZU01000028), *Dickeya dianthicola* NCPPB 453 (NZ_AOOB01000052), *E. ictaluri* ATCC 33202 (NZ_AFJI01000117), *E. tarda* ATCC 15947 (NZ_BANW01000001), and *E. coli* DSM 30083 (NZ_KK583188). These findings could imply that pathogenic bacteria may avoid the negative effects of plant-derived compounds on their cells and continue to remain in their host niches.

Our analysis showed that *Lactobacillus* genomes consisted of higher number of genes involved in the metabolism of different ANFs (12 and five genomes can metabolize on five and six ANFs, respectively) compared to other genomes (maximal numbers are four ANFs). This result is in agreement with a previous study that LABs were isolated from fermented breadfruit and cowpea flours and were identified as *Lacobacillus* (14 different species), *Leuconostoc messenteroide*, and *Pediococcus acidilactis* (Ojokoh et al., 2013). The most dominant isolates in the fermentation were *L. plantarum*. These LABs not only might affect the reduction in the entire antinutrients including hydrogen cyanide, oxalate, and phytate but also decrease in the population of opportunistic pathogenic species that may be contaminated through the fermentation progress.

In line with previous studies in animal feed (e.g., Qian et al., 2018; de Oliveira et al., 2022), our findings indicated that *Lactobacillus* can help in the nutritional value of converted plant-based protein while lowering antinutrient content. By fermenting grain flour at 37°C for 24 h with *L. acidophilus*, phytic acid and polyphenol content may be lowered (Binita R, Khetarpaul et al., 1997), whereas Ojokoh et al. (2013) demonstrated that the overall antinutrient qualities of plant-based diet were significantly reduced after a day of fermentation. In particular, *L. plantarum*-produced α-galatosidase utilized as a starting culture considerably decreased antinutrients such as trypsin inhibitor (69% reduction), protease inhibitor (30%), phytate (60% reduction), and tannin (72% reduction) present in sorghum (observed at 120 h) (Adeyemo et al., 2016). Meanwhile, 58% of trypsin inhibitor, 40% of protease inhibitor, 70% of phytate, and 56% of tannin were decreased after 120 h of utilizing brevis as a starting culture.

In addition to eliminating the pathogenic strains through virulence and antimicrobial resistant genes detection, pan-genome analysis may help to maximize in the reduction in ANFs in plant-based food by treatment combination between strain-dependent *B. subtilis* and *Lactobacillus* species. Using omics approach, we can predict species and/or strains that may merge together in fermentation to maximize removal of antinutrient factors. For example, the mixture of three strains including *L. fabifermentans* DSM 21115 (NZ_AYGX02000079), *L. brevis* ATCC_14869 (NZ_AZCP01000009), and *B. subtilis* SRCM103689 (NZ_CP035391) (or strain SX01705 (NZ_CP022287) may result in highest number of genes (28 genes) involved in the improvement of eight antinutrient factors such as phytase, *Myo*-inositol, lectin, tannase, phenolic compound, trypsin, α-Gal, and saponin-degrading enzymes (**Supplementary Table S5**). Our findings may provide an information for the previous study that measured both *B. subtilis* and *Lactobacillus* species after fermentation of breadfruit and cowpea blend (Ojokoh et al., 2013), and *B. subtilis* may participate in removing antinutritional content together with the presence of *Lactobacillus* species.

## Conclusions

Plant-derived materials may provide health benefits against some diseases, but they may include several ANFs with toxic potential. Among the traditional methods and technologies centered on reducing the levels of these factors, fermentation based on unique microorganism (ensiling with LAB) that consists measurable activities of antinutrient removal is an alternative, maximizing the number of degrading factors and is time consuming. Based on four pan-genome analyses, genes encoding for production/enzyme participating in metabolism and/or brake ANFs were extracted and compared. *Lactobacillus* species have higher numbers of metabolism (7) involved in different types of ANFs than *B. subtilis* (4); however, phytase enzyme was only detected in the genome of *B. subtilis* (**Supplementary Table S5**).

We hypothesize that a combination of two *Lactobacillus* strains (DSM 21115 and ATCC_14869) with *B. subtilis* SRCM103689 in fermentation was suggested to produce maximum yields and type of enzymes/productions that may highly reduce the concentration of ANFs. Our study, therefore, provides new insights into bacterial strain screening approaches for maximizing nutritional value in food. Further investigations on gene numbers and repertories correlated with different ANFs will help in clarifying the efficiency of time consumption and food qualities.

## Data availability statement

The original contributions presented in the study are included in the article/**Supplementary Material**. Further inquiries can be directed to the corresponding author.

## Author contributions

TN designed the research. HP and TN were involved in the conceptualization and contributed to the software. TN was in charge of acquiring funds and data analysis. HP discussed the findings and drafted the manuscript. DHK helped with editing and proofreading the final version. All authors contributed to and approved the final version of the manuscript.

## Supplementary material

Supplementary Table 1 Dispensable genes potentially involved in metabolism of ANFs of bacterial species belonged to *Enterobacteriaceae*. The binary analysis of each ANF-related genes was separated into sub-sheets.

Supplementary Table 2 Dispensable genes potentially involved in metabolism of ANFs of bacterial species belonged to *Lactobacillaceae*. The binary analysis of each ANF-related genes was separated into sub-sheets.

Supplementary Table 3 Dispensable genes potentially involved in metabolism of ANFs of *Bacillus subtilis*. The binary analysis of each ANF-related genes was separated into sub-sheets.

Supplementary Table 4 Pediococcus: Dispensable genes potentially involved in metabolism of ANFs of *Pediococcus* species. The binary analysis of each ANF-related genes was separated into sub-sheets.

Supplementary Table 5 Comparative analysis of ANFs metabolic capability between species/strains in the genus/family, and the results of prospective strains.
